# Supplementary figures and images for: Balanced state of networks of winner-take-all units
Source: PLoS Comput Biol. 2025 Jun 11;21(6):e1013081. doi: 10.1371/journal.pcbi.1013081 (PMC12157085; doi:10.1371/journal.pcbi.1013081)

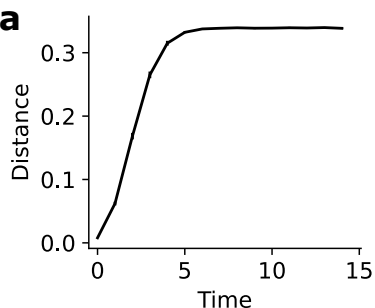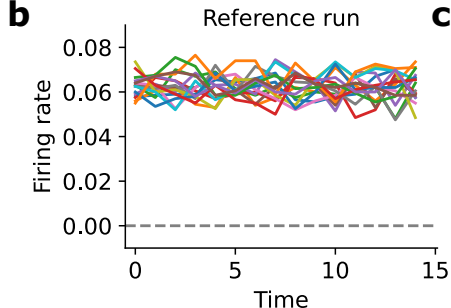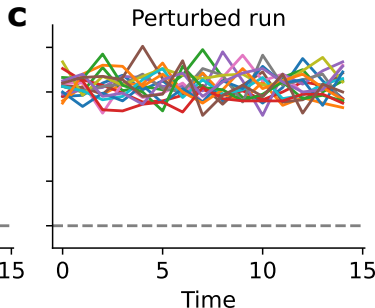

Supplement: S1 Fig — a. Root-mean-square distance between the microscopic network state ({𝐲dt}) vs time since a single spike-swap perturbation is introduced. Parameters used were: D = 16, N = 2000; μJ(d,d′)=0∀d,d′; σJ2(d,d′)=1∀d,d′. b-c. Firing rates corresponding to the reference run (b) vs the perturbed run (c, in which the spike swap is introduced at t=0). (PDF) [file pcbi.1013081.s001.pdf]

**a**

Uniform state stability  
(mean-driven)

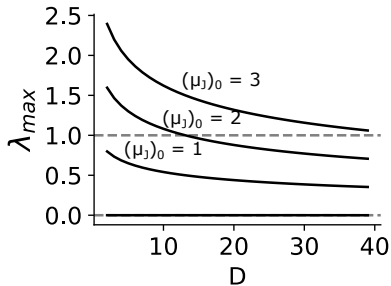**b**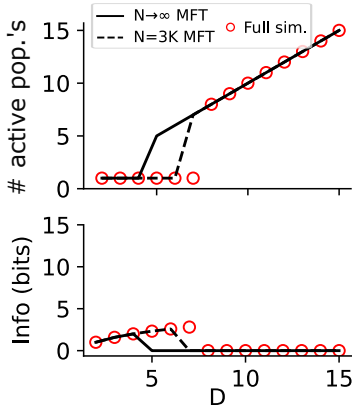

Supplement: S3 Fig — a. Maximum eigenvalue λmax of the Jacobian of the N→∞ mean-field dynamics evaluated at the uniform distribution 1/D as a function of D and (μJ)0. b. Number of active populations (top) or equivalently information content (bottom) for the mean-driven network ((μJ)0=1.5). Solid lines are theory; circles are full simulation results (N = 3000). (PDF) [file pcbi.1013081.s003.pdf]

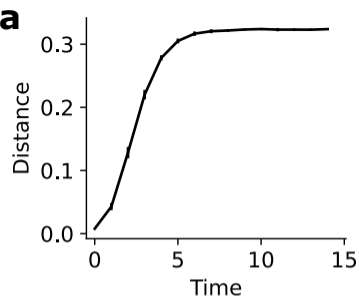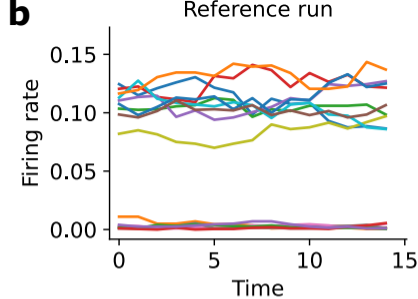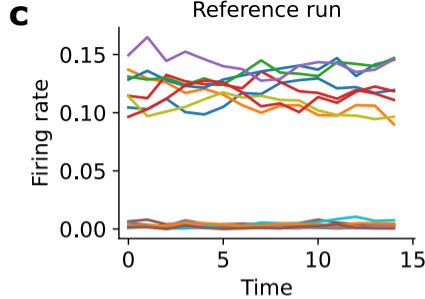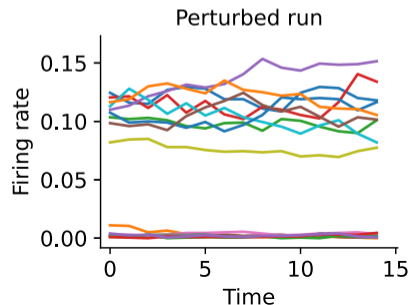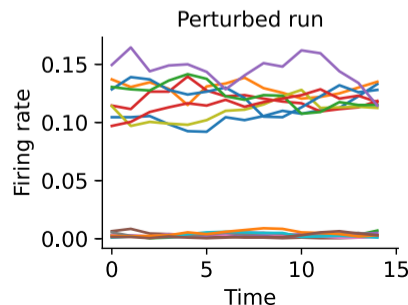

Supplement: S4 Fig — a. Root-mean-square distance between the microscopic network state ({𝐲dt}) vs time since a single spike-swap perturbation is introduced, in the fluctuation-driven multistable network. Trace and error bars show mean and standard deviation, respectively, over 10 trials. Parameters used were N = 2000, D = 16, (σJ)0=1, (σJ)1=0.05. b. Evolution of firing rates for a reference simulation and a perturbed simulation (created by a single spike swap, relative to the reference network, at time 0). c. As in b, but for another trial. (PDF) [file pcbi.1013081.s004.pdf]

**a**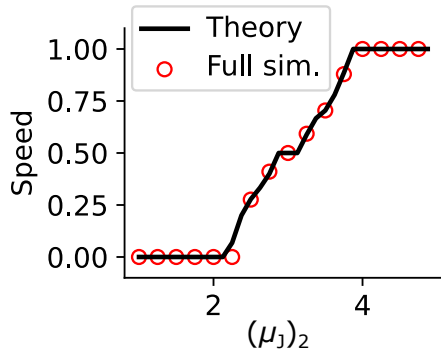**b**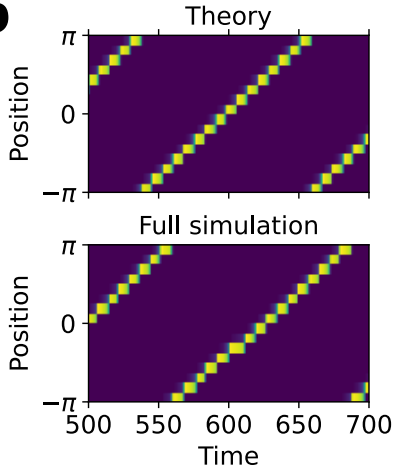**c**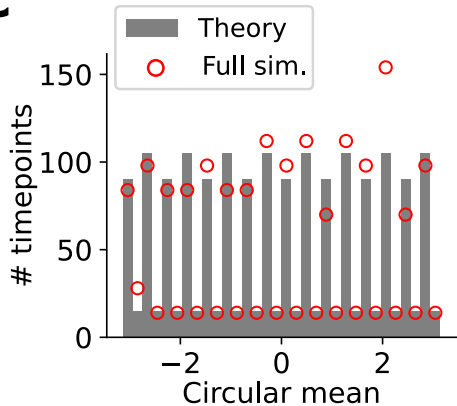

Supplement: S6 Fig — a. Theoretical predictions and full simulations (N=2000,(μJ)0=3) of sequence propagation speed in the mean-driven network. b. Heatmaps of the evolution of macroscopic network activity (firing rates) in the mean-driven sequence-generating network, via either the N→∞ theory or a full simulation ((μJ)0=3,(μJ)2=2.3,N=2500). c. Histogram of circular means of macroscopic activity distribution in the mean-driven sequence-generating network. (PDF) [file pcbi.1013081.s006.pdf]

**a**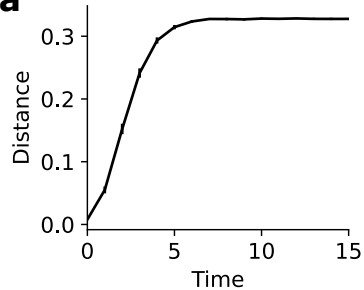**b**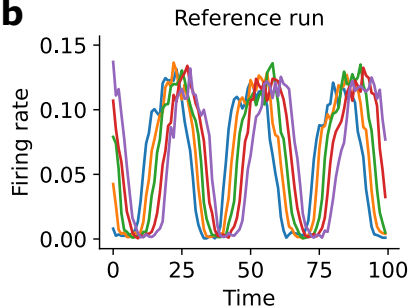**c**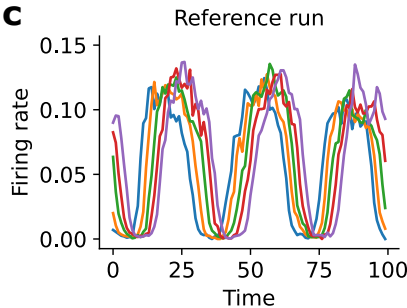

Supplement: S7 Fig — a. Root-mean-square distance between the microscopic network state ({𝐲dt}) vs time since a single spike-swap perturbation is introduced, in the fluctuation-driven sequence-generating network. Trace and error bars show mean and standard deviation, respectively, over 10 trials. Parameters used were N = 2000, D = 16, (σJ)0=10, (σJ)1=1, (σJ)2=10. b. Evolution of firing rates for a reference simulation and a perturbed simulation (created by a single spike swap, relative to the reference network, at time 0). c. As in b, but for another trial. (PDF) [file pcbi.1013081.s007.pdf]
