# Supplementary material for: Balanced state of networks of winner-take-all units
Source: PLoS Comput Biol. 2025 Jun 11;21(6):e1013081. doi: 10.1371/journal.pcbi.1013081 (PMC12157085; doi:10.1371/journal.pcbi.1013081)
Supplement: S2 Fig — a. Schematic of network weights for mean-driven multistability. b. Mean-field simulations (N→∞) of firing rate dynamics for 6 different (μJ)0. c. Example firing rate dynamics and spike trains from a full simulation of the mean-driven multistable network ((μJ)0=3, N = 3000, D = 16). (PDF) [file pcbi.1013081.s002.pdf]

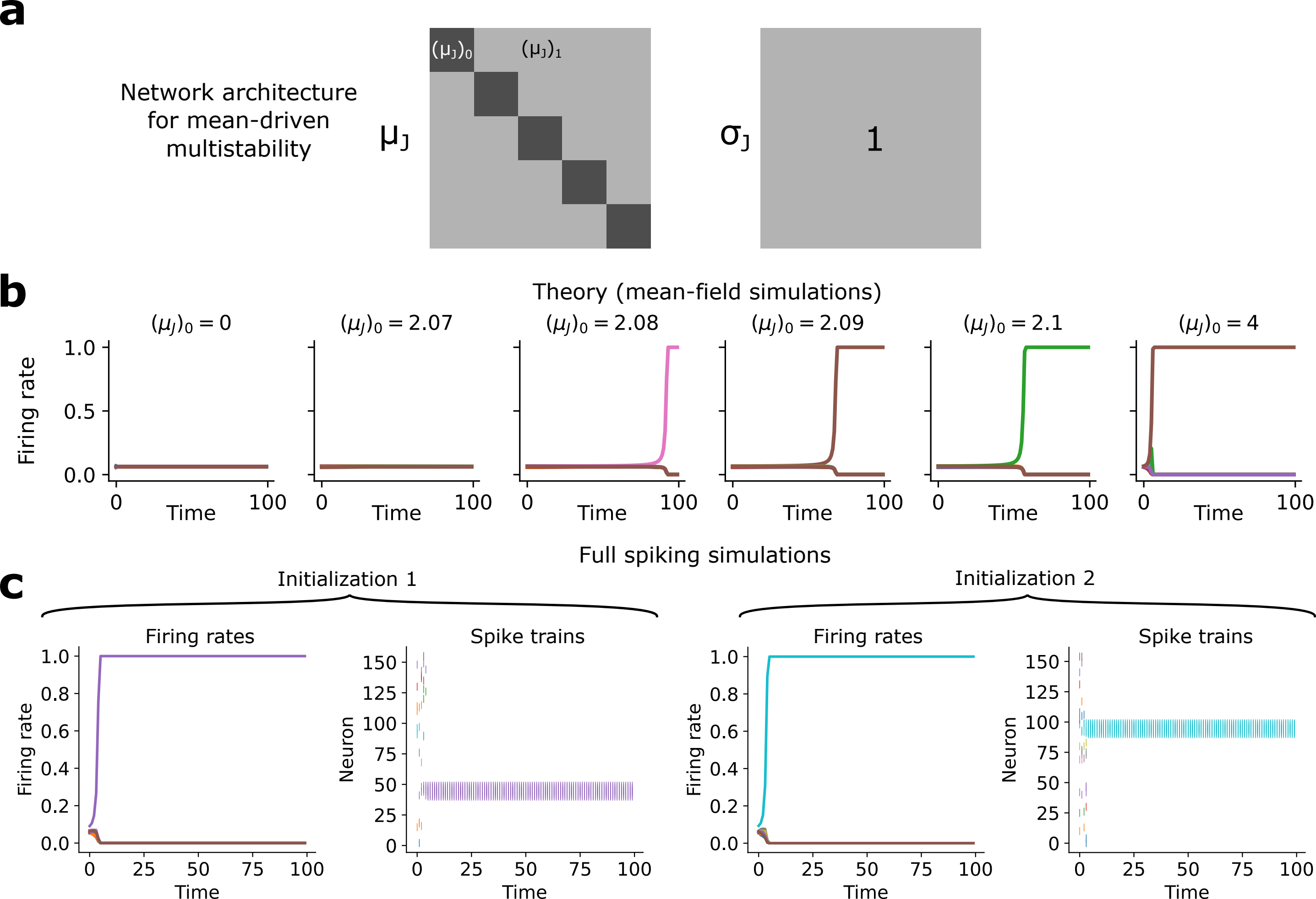
a
Network architecture for mean-driven multistability
μ_J
$(\mu_J)_0$
$(\mu_J)_1$
σ_J
1
b
Theory (mean-field simulations)
$(\mu_J)_0 = 0$
$(\mu_J)_0 = 2.07$
$(\mu_J)_0 = 2.08$
$(\mu_J)_0 = 2.09$
$(\mu_J)_0 = 2.1$
$(\mu_J)_0 = 4$
Firing rate
Time
Full spiking simulations
c
Initialization 1
Initialization 2
Firing rates
Spike trains
Neuron
Time
