# Supplementary material for: Balanced state of networks of winner-take-all units
Source: PLoS Comput Biol. 2025 Jun 11;21(6):e1013081. doi: 10.1371/journal.pcbi.1013081 (PMC12157085; doi:10.1371/journal.pcbi.1013081)
Supplement: S5 Fig — a. Schematic of network weights for mean-driven sequence generation. b. N→∞ mean-field simulations of firing rate dynamics in mean-driven sequence generation, for 5 different values of (μJ)2, with (μJ)0=3, and D = 16. c. Population firing rates and spike trains from an example full network simulation with (μJ)0=(μJ)2=3,(σJ)0=(σJ)1=1 and N=2000,D=16. (PDF) [file pcbi.1013081.s005.pdf]

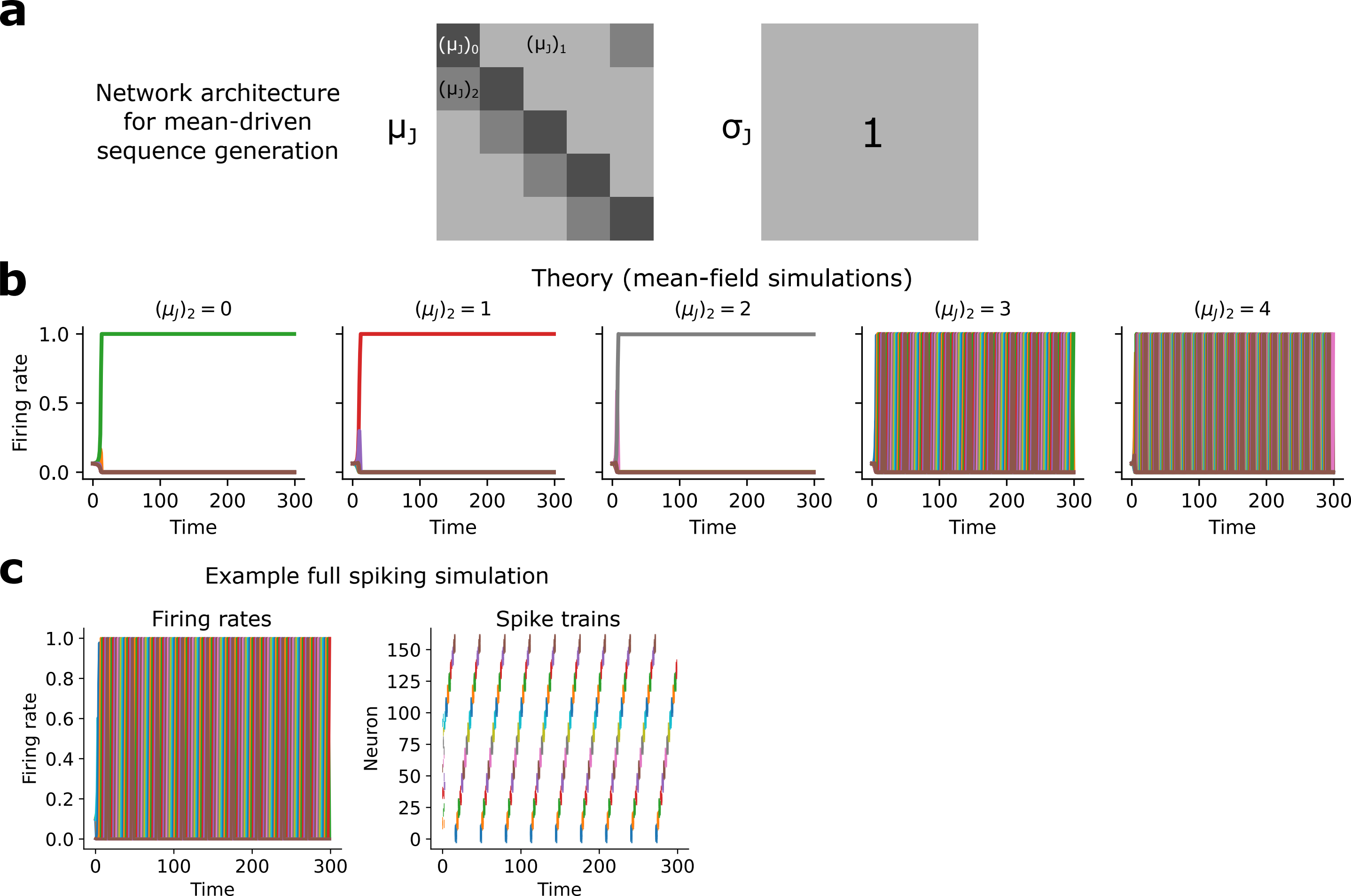
a
Network architecture
for mean-driven
sequence generation
μJ
(μJ)0
(μJ)1
(μJ)2
σJ
1
b
Theory (mean-field simulations)
$(\mu_J)_2 = 0$
$(\mu_J)_2 = 1$
$(\mu_J)_2 = 2$
$(\mu_J)_2 = 3$
$(\mu_J)_2 = 4$
Firing rate
1.0
0.5
0.0
0
100
200
300
Time
c
Example full spiking simulation
Firing rates
Spike trains
Firing rate
1.0
0.8
0.6
0.4
0.2
0.0
Neuron
150
125
100
75
50
25
0
Time
