# Supplementary material for: Balanced state of networks of winner-take-all units
Source: PLoS Comput Biol. 2025 Jun 11;21(6):e1013081. doi: 10.1371/journal.pcbi.1013081 (PMC12157085; doi:10.1371/journal.pcbi.1013081)
Supplement: S1 Appendix — (PDF) [file pcbi.1013081.s008.pdf]

S1 Appendix: Full derivation of mean-field dynamics for the firing rates

Here we derive the mean-field equations (Eq 5-10 in the main text) for the time evolution of the macroscopic network state $\mathbf{r}^t$, i.e. the vector of firing rates of the D co-tuned populations, in the spiking network limit ($g \to \infty$).

Number of neurons spiking in each population

Recall that $\mathbf{r}^t = \mathbf{P}^t$, the categorical distribution over which of the neurons in the representative unit i^* spikes at time t. Here we focus on $\mathbf{P}^t$, to emphasize that the object of study is a discrete probability distribution, even though this quantity is identical to the firing rate vector $\mathbf{r}^t$. Let the state of unit i at time t be $\mathbf{y}_i^t \in \{\mathbf{1}_1, \ldots, \mathbf{1}_D\}$, where $\mathbf{1}_d$ is the 1-hot vector whose d-th element equals 1. The number of units in state $\mathbf{1}_d$ at time t we define as

$$n_d^t = \sum_i 1[\mathbf{y}_i^t = \mathbf{1}_d] \qquad \text{with} \qquad \sum_d n_d^t = N \quad \forall t \qquad \text{and} \qquad \mathbf{n}^t \equiv [n_1^t, \ldots, n_D^t]. \tag{1}$$

Although the network is deterministic, we assume it is chaotic enough to be studied statistically. To this end we treat each unit as statistically identical, with $\mathbf{y}_i^t \sim \mathbf{P}^t$.

Input distribution

We first derive the distribution of the inputs. The inputs x_{id}^t are approximately random variables given by

$$x_{id}^t = \sum_{d',j} J_{ij}^{dd'} y_{jd'}^{t-1} + u_{id}^t \tag{2}$$

where $J_{ij}^{dd'}$ are the (quenched) synaptic connections, with

$$J_{ij}^{dd'} \sim \mathcal{N}\left(\mu_J(d,d')\frac{D}{N}, \sigma_J^2(d,d')\frac{D}{N}\right) \tag{3}$$

and u_{id}^t the external input to the d-th neuron in unit i. We moreover assume that the previous states are i.i.d. with

$$\mathbf{y}_i^{t-1} \sim \mathbf{P}^{t-1}. \tag{4}$$

Treating x_{id}^t as Gaussian,

$$x_{id}^t \sim P(x_{id}^t) \equiv \mathcal{N}\left(\mu_d, \sigma_d^2\right), \tag{5}$$

computing the mean μ_d and variance σ_d^2 completely determine the distribution over the inputs x_{id}^t. Let $Y^{t-1} \equiv \{y_{jd}^{t-1}\}$ denote the microscopic (spiking) state of the network at time $t-1$. To compute the input means and variances, μ_d and σ_d^2, we treat Y^{t-1} as a random variable (given the previous firing rate vector $\mathbf{r}^{t-1}$):

$$\mu_d \equiv \left\langle x_{id}^t \right\rangle_Y = \left\langle \sum_{d',j} J_{ij}^{dd'} y_{jd'}^{t-1} + u_{id}^t \right\rangle_Y \tag{6}$$

$$\sigma_d^2 \equiv \mathrm{Var}\left[x_{id}^t\right]_Y = \mathrm{Var}\left[\sum_{d',j} J_{ij}^{dd'} y_{jd'}^{t-1} + u_{id}^t\right]_Y \tag{7}$$

To handle the distribution over the states of the units at time $t-1$ we expand

$$P(Y^{t-1}) = \sum_{\mathbf{n}^{t-1}} P(Y^{t-1}|\mathbf{n}^{t-1}) P(\mathbf{n}^{t-1}). \tag{8}$$

If we assume a large number of units, then $P(\mathbf{n}^{t-1})$ will be highly peaked at its mean $\mathbf{n}^{t-1} = N\mathbf{P}^{t-1}$, so we can replace the sum with the single value

$$P(Y^{t-1}) \approx P(Y^{t-1}|N\mathbf{P}^{t-1}) \tag{9}$$

i.e. a distribution in which the total number of units in each state $\mathbf{1}_d$ is fixed and all the remaining randomness corresponds to which units are in which states.

Averaging over the quenched disorder

Our equations 6-7 for μ_d and σ_d^2 are still functions of the quenched disorder, i.e. the synaptic weights $J_{ij}^{dd'}$. We are interested in the case, however, in which the macroscopic network activity is largely independent of the specific instantiation of the quenched disorder. That is, given the previous network state $Y^{t-1} = \{y_{jd'}^{t-1}\}$ (which we treat as random and conditional on $\mathbf{P}^{t-1}$) we would like μ_d and σ_d^2 to be roughly the same for all typical instantiations of $J_{ij}^{dd'}$, such that we can approximate

$$\mu_d \approx \langle \mu_d \rangle_J = \left\langle \left\langle \sum_{d',j} J_{ij}^{dd'} y_{jd'}^{t-1} + u_{id}^t \right\rangle_Y \right\rangle_J = \left\langle \left\langle \sum_{d',j} J_{ij}^{dd'} y_{jd'}^{t-1} \right\rangle_Y \right\rangle_J + \mathrm{E}[u_{id}^t] \tag{10}$$

$$\sigma_d^2 \approx \langle \sigma_d^2 \rangle_J = \left\langle \mathrm{Var}\left[\sum_{d',j} J_{ij}^{dd'} y_{jd'}^{t-1} + u_{id}^t\right]_Y \right\rangle_J = \left\langle \mathrm{Var}\left[\sum_{d',j} J_{ij}^{dd'} y_{jd'}^{t-1}\right]_Y \right\rangle_J + \mathrm{Var}[u_{id}^t] \tag{11}$$

Recalling that the $J_{ij}^{dd'}$ are i.i.d. given d, d', it is helpful to write

$$\mu_d \approx \left\langle \left\langle \sum_j J_{ij}^{d1} y_{j1}^{t-1} \right\rangle_Y \right\rangle_J + \cdots + \left\langle \left\langle \sum_j J_{ij}^{dD} y_{jD}^{t-1} \right\rangle_Y \right\rangle_J + \mathrm{E}[u_{id}^t] \tag{12}$$

$$\sigma_d^2 \approx \left\langle \mathrm{Var}\left[\sum_j J_{ij}^{d1} y_{j1}^{t-1}\right]_Y \right\rangle_J + \cdots + \left\langle \mathrm{Var}\left[\sum_j J_{ij}^{dD} y_{jD}^{t-1}\right]_Y \right\rangle_J + \mathrm{Var}[u_{id}^t]. \tag{13}$$

To compute these, let

$$\Omega_d(Y^{t-1}) \equiv \{j \in \{1, \ldots, N\} | y_{jd}^{t-1} = 1\} \tag{14}$$

be the set of units in the d-th state $\mathbf{1}_d$ at time $t-1$. Then

$$\sum_j J_{ij}^{dd'} y_{jd'}^{t-1} = \sum_{j \in \Omega_{d'}} J_{ij}^{dd'} \tag{15}$$

The d'-th term of the mean μ_d (Eq 12) is then

$$\left\langle \left\langle \sum_j J_{ij}^{dd'} y_{jd'}^{t-1} \right\rangle_Y \right\rangle_J = \left\langle \left\langle \sum_{j\in\Omega_{d'}} J_{ij}^{dd'} \right\rangle_Y \right\rangle_J = \left\langle \sum_{j\in\Omega_{d'}} \langle J_{ij}^{dd'} \rangle_J \right\rangle_Y = n_{d'}^{t-1} \langle J_{ij}^{dd'} \rangle_J. \tag{16}$$

Thus

$$\begin{aligned} \mu_d \approx & \mu_J(d,1)\frac{D}{N} n_1^{t-1} + \cdots + \mu_J(d,D)\frac{D}{N} n_D^{t-1} + \mathrm{E}[u_{id}^t] \\ =& D\sum_{d'} \mu_J(d,d')\frac{n_{d'}^{t-1}}{N} + \mathrm{E}[u_{id}^t]. \end{aligned} \tag{17}$$

The variance is computed in a similar fashion:

$$\begin{aligned} \left\langle \mathrm{Var}\left[\sum_j J_{ij}^{dd'} y_{jd'}^{t-1}\right]_Y \right\rangle_J &= \left\langle \mathrm{Var}\left[\sum_{j\in\Omega_{d'}} J_{ij}^{dd'}\right]_Y \right\rangle_J \\ &= \left\langle \left\langle \left(\sum_{j\in\Omega_{d'}} J_{ij}^{dd'}\right)^2 \right\rangle_Y - \left\langle \sum_{j\in\Omega_{d'}} J_{ij}^{dd'} \right\rangle_Y^2 \right\rangle_J \end{aligned} \tag{18}$$

$$= \left\langle \left\langle \sum_{j\in\Omega_{d'}} (J_{ij}^{dd'})^2 + \sum_{j\in\Omega_{d'}} \sum_{j'\neq j\in\Omega_{d'}} J_{ij}^{dd'} J_{ij'}^{dd'} \right\rangle_Y - \left\langle \sum_{j\in\Omega_{d'}} J_{ij}^{dd'} \right\rangle_Y^2 \right\rangle_J \tag{19}$$

$$= \left\langle \sum_{j\in\Omega_{d'}} \langle (J_{ij}^{dd'})^2 \rangle_J + \sum_{j\in\Omega_{d'}} \sum_{j'\neq j\in\Omega_{d'}} \langle J_{ij}^{dd'} \rangle_J \langle J_{ij'}^{dd'} \rangle_J \right\rangle_Y - \left\langle \left\langle \sum_{j\in\Omega_{d'}} J_{ij}^{dd'} \right\rangle_Y^2 \right\rangle_J \tag{20}$$

$$\begin{aligned} &= \left\langle n_{d'}^{t-1} \left(\mathrm{Var}\left[J_{ij}^{dd'}\right]_J + \langle J_{ij}^{dd'} \rangle_J^2 \right) \right\rangle_Y \\ &+ \left\langle n_{d'}^{t-1} \left(n_{d'}^{t-1} - 1\right) \langle J_{ij}^{dd'} \rangle_J^2 \right\rangle_Y - \left\langle \left\langle \sum_{j\in\Omega_{d'}} J_{ij}^{dd'} \right\rangle_Y^2 \right\rangle_J \end{aligned} \tag{21}$$

$$= n_{d'}^{t-1} \left(\mathrm{Var}\left[J_{ij}^{dd'}\right]_J + \langle J_{ij}^{dd'} \rangle_J^2 \right) + n_{d'}^{t-1} \left(n_{d'}^{t-1} - 1\right) \langle J_{ij}^{dd'} \rangle_J^2 - \left\langle \left\langle \sum_{j\in\Omega_{d'}} J_{ij}^{dd'} \right\rangle_Y^2 \right\rangle_J \tag{22}$$

$$= n_{d'}^{t-1} \sigma_J^2(d,d') \frac{D}{N} + \left(n_{d'}^{t-1}\right)^2 \langle J_{ij}^{dd'} \rangle^2 - \left\langle \left\langle \sum_{j\in\Omega_{d'}} J_{ij}^{dd'} \right\rangle_Y^2 \right\rangle_J. \tag{23}$$

To estimate the last term we approximate the distribution

$$P\left(\{y_{jd}^{t-1}\}\right) \approx \prod_j P(y_{jd}^{t-1}) \qquad \text{while keeping} \qquad \langle y_{jd}^{t-1} \rangle_Y = \frac{n_d^{t-1}}{N}. \tag{24}$$

That is, instead of randomly assigning exactly n_d^{t-1} units to state $\mathbf{1}_d$ we treat each unit as independent such that the mean number of units assigned to state $\mathbf{1}_d$ is n_d^{t-1}. Then

$$\left\langle \sum_{j\in\Omega_{d'}} J_{ij}^{dd'} \right\rangle_Y = \left\langle \sum_{j=1}^{N} J_{ij}^{dd'} y_{id'}^{t-1} \right\rangle_Y = \sum_{j=1}^{N} J_{ij}^{dd'} \langle y_{id'}^{t-1} \rangle_Y = n_{d'}^{t-1} \frac{1}{N} \sum_{j=1}^{N} J_{ij}^{dd'} \tag{25}$$

so that

$$\begin{aligned} \left\langle \left\langle \sum_{j\in\Omega_{d'}} J_{ij}^{dd'} \right\rangle_Y^2 \right\rangle_J &= \left(n_{d'}^{t-1}\right)^2 \left\langle \left(\frac{1}{N} \sum_{j-1}^{N} J_{ij}^{dd'} \right)^2 \right\rangle_J \\ &= \left(n_{d'}^{t-1}\right)^2 \left\langle J_{ij}^{dd'} \right\rangle_J^2 (1 + \mathcal{O}(1/N)) \approx \left(n_{d'}^{t-1}\right)^2 \left\langle J_{ij}^{dd'} \right\rangle_J^2. \end{aligned} \tag{26}$$

Thus, the last two terms in Eq 23 cancel, and we are left with

$$\left\langle \mathrm{Var}\left[\sum_j J_{ij}^{dd'} y_{jd'}^{t-1} \right]_Y \right\rangle_J \approx n_{d'}^{t-1} \sigma_J^2(d,d') \frac{D}{N} \tag{27}$$

i.e. just the variance of $n_{d'}^{t-1}$ samples of $J_{ij}^{dd'}$, matching our intuition.

Finally, combining the D terms in the variance expression (Eq 13) we arrive at

$$\begin{aligned} \sigma_d^2 \approx& \sigma_J^2(d,1) \frac{D}{N} n_1^{t-1} + \cdots + \sigma_J^2(d,D) \frac{D}{N} n_D^{t-1} + \mathrm{Var}[u_{id}^t] \\ =& D \sum_{d'} \sigma_J^2(d,d') \frac{n_{d'}^{t-1}}{N} + \mathrm{Var}[u_{id}^t]. \end{aligned} \tag{28}$$

which become

$$\mu_d \approx D \sum_{d'} \mu_J(d,d') P_d^{t-1} + \mathrm{E}[u_{id}^t] \tag{29}$$

$$\sigma_d^2 \approx D \sum_{d'} \sigma_J^2(d,d') P_d^{t-1} + \mathrm{Var}[u_{id}^t] \tag{30}$$

when $N \to \infty$, recapitulating Eq. 9-10 in the main text, since $\mathbf{P}^t = \mathbf{r}^t$.

Winner-take-all competition

Given the distribution over x_{id}^t, the probability of a unit i being in state $\mathbf{y}_i^t = \mathbf{1}_d$ is

$$P_d^t \equiv P(\mathbf{y}_i^t = \mathbf{1}_d) = P(x_{id}^t \geq x_{i1}^t, \ldots, x_{id}^t \geq x_{iD}^t), \tag{31}$$

i.e. the unit takes the state $\mathbf{1}_d$ if its d-th input is greater than all the rest, reflecting the WTA nonlinearity. Expanding the previous equation we have

$$P_d^t = \int_{-\infty}^{\infty} dx P(x_{id}^t = x) \prod_{d'\neq d} P(x_{id}^t \geq x_{id'}^t | x_{id}^t = x) \tag{32}$$

where we have removed the $d' = d$ term from the product since $P(x_{id}^t \geq x_{id}^t | x_{id}) = 1$. All terms here, however, are Gaussians (with the terms inside the product given by cumulative Gaussians), so that we can write

$$P_d^t | \mathbf{P}^{t-1} = \int dx \mathcal{N}(x; \mu_d, \sigma_d^2) \prod_{d'\neq d} \Phi(x; \mu_{d'}, \sigma_{d'}^2) \tag{33}$$

which combined with Eq. 29-30 specifies the complete update rule for $\mathbf{P}^t$.
