# Supplementary material for: Balanced state of networks of winner-take-all units
Source: PLoS Comput Biol. 2025 Jun 11;21(6):e1013081. doi: 10.1371/journal.pcbi.1013081 (PMC12157085; doi:10.1371/journal.pcbi.1013081)
Supplement: S2 Appendix — (PDF) [file pcbi.1013081.s009.pdf]

S2 Appendix: Approximate simulations for large networks

For full spiking simulations for very large networks (Fig 5c, 8e, 9b-e), we store only a single $N \times N$ connectivity matrix J_0 in memory, then scale, shift, and permute it at runtime for each (d, d'). This procedure approximates the storage and access of the full 4-tensor $\{J_{ij}^{dd'}\}$.

We first sample J_0 via:

$$(J_0)_{ij} \sim \mathcal{N}(0, 1). \tag{1}$$

Next we rescale J_0 so that it has exactly zero mean and unit variance. For each block (d, d') we then sample a realistic sample mean $\tilde{\mu}_J$ and variance $\tilde{\sigma}_J^2$ of the weights via

$$\tilde{\mu}_J(d, d') \sim \mathcal{N}\left(\mu_J(d, d')\frac{D}{N}, \sigma_J^2(d, d')\frac{D}{N} \times \frac{1}{N^2}\right) \tag{2}$$

$$\tilde{\sigma}_J^2(d, d') \times \frac{N^2 - 1}{\sigma_J^2(d, d')D/N} \sim \chi^2\left(N^2 - 1\right). \tag{3}$$

where χ^2 is the chi-squared distribution with $N^2 - 1$ degrees of freedom. This produces realistic sample means and variances of the weights across the blocks; (if we simply scaled and shifted J_0 to make each block then the within-block sample means and variances of the weights would be equal across all blocks).

We then let

$$J^{dd'} = P_{row}^{dd'}\left(\tilde{\sigma}_J(d, d')J_0 + \tilde{\mu}_J(d, d')\hat{1}\right)P_{col}^{dd'}. \tag{4}$$

where $P_{row}^{dd'}$ and $P_{col}^{dd'}$ are quenched $N \times N$ random permutation matrices that rearrange the rows and columns of the scaled, shifted J_0, and $\hat{1}$ is the $N \times N$ matrix of 1's. For an N-dimensional vector $y_{:,d'}^{t-1}$ of the activities of neurons with tuning d' at time $t - 1$, we then have

$$J^{dd'}y_{:,d'}^{t-1} = P_{row}^{dd'}\left(\tilde{\sigma}_J(d, d') \times J_0 + \tilde{\mu}_J(d, d')\hat{1}\right)P_{col}^{dd'}y_{:,d'}^{t-1}. \tag{5}$$

This can be computed essentially as efficiently as a single matrix multiplication because both applications of the permutation operators can be implemented via logical masking.
