# Supplementary material for: Balanced state of networks of winner-take-all units
Source: PLoS Comput Biol. 2025 Jun 11;21(6):e1013081. doi: 10.1371/journal.pcbi.1013081 (PMC12157085; doi:10.1371/journal.pcbi.1013081)
Supplement: Attachment — Submitted filename: response_to_reviews.pdf [file pcbi.1013081.s010.pdf]

Response to Reviews for:
“Balanced state of networks of winner-take-all units”

We thank the Reviewers for the helpful feedback and comments about our study, which we have incorporated into a revised and improved manuscript. The major changes we have made include (1) a more careful introduction to the notions of balance and asynchronous irregular activity, as well as the meaning of “balance” in the network proposed in this study, as suggested by Reviewer 1; (2) a clearer description of the motivation/approach/results of the final section of the study about generating heterogeneous firing rate dynamics with the model, as suggested by Reviewer 2; (3) incorporating references/discussion of related work suggested by the Reviewers; and (4) making the figures easier to read—in particular, we have increased the font sizes on many of the axis labels as well as split the three largest figures (previously Fig 4, 5, 6) into Figs 4-5, Figs 6-7, and Figs 8-9; which we expect will make them clearer. Additional minor changes are noted throughout our response. We have also made various minor line edits throughout the text for increased clarity.

Below we respond point-by-point to the reviews. Reviewer comments are given in **violet**, our responses in **black**, and quotations from the revised manuscript in **magenta** (citations are abbreviated here but are in full in the manuscript). All changes to the text itself are marked in **magenta** in the attached manuscript file.

Reviewer #1:
This manuscript presents a novel model of interacting populations of winner take all networks. The authors present a mathematical analysis of the networks in the high gain limit and show that the networks can produce asynchronous irregular spiking activity, sequence generation, and other common features of neural activity in the brain. The model is interesting and, as far as I am aware, novel. Given the novelty of this approach, the authors do a good job of laying the groundwork for future work on developing these models, which could hold promise for better understanding neural circuit dynamics.

Thank you very much for the comment and accurate summary of the study.

I only have a handful of minor comments:

1) The title of the manuscript and the body of the manuscript (esp the Introduction) mention "balance" but it seems that the authors are using this term at least in part to mean asynchronous-irregular activity. In traditional network models (e.g., integrate-and-fire or binary network models), excitatory-inhibitory balance often coincides with asynchronous-irregular activity and EI balance was historically studied as a mechanism to generate asynchronous-irregular activity. However, they do not always coincide and they are fundamentally different phenomena. Network models can exhibit EI balance without asynchronous-irregular activity or asynchronous-irregular activity without EI balance. The authors mention some notion of "balance" for example when they write (line 159) "the balance of inputs from different co-tuned populations" but this seems fundamentally different from excitatory-inhibitory balance, which is what the term "balance" normally refers to in the field. If there is a direct connection between these two notions of balance, the authors should clarify it. If not, the authors should avoid conflating the two (e.g., (line 32) "we present a generalization of the classic balanced state"; in what sense is their model a generalization?). In general, I think that the relationships between asynchronous-irregular activity, excitatory-inhibitory balance, and whatever notion of balance their models exhibit should be made more clear. And the authors should avoid statements that conflate these phenomena. I realize that this is a somewhat open-ended suggestion and I will not nitpick exactly how the authors choose to address it. This is more of a suggestion.

Thank you for the suggestion. We apologize for the imprecise description of these concepts in the original manuscript. The Reviewer is correct that balance does not always coincide with the asynchronous irregular regime, even though they often coexist in spiking network models of cortical circuits.

To clarify these points and concepts throughout our manuscript we have now made several edits to our Introduction:

- The relevant section of the first paragraph of the Introduction now introduces asynchronous irregular activity explicitly (LINES 5-13):

  …we still lack a comprehensive account of the role of the microscopic activity of the neurons involved, which in many brain areas and conditions manifests as sparse spike trains with high temporal irregularity and weak correlations (Funahashi 1989; Softky 1993; Holt 1996; Shadlen 1998; Compte 2003; Ahmadian 2021). This Poisson process-like spiking regime, termed the *asynchronous irregular* regime, has been most commonly observed in cerebral cortex of awake mammals, for example in primates performing working memory tasks (Funahashi 1989; Compte 2003), and has motivated substantial theoretical work investigating its origin and function (Van Vreeswijk 1996; Van Vreeswijk 1998; Brunel 2000; Vogels 2009; Vogels 2011; Monteforte 2012; Baker 2020; Sanzeni 2020; Khajeh 2022; Ahmadian 2021). Reconciling the asynchronous irregular regime with more complex, flexible collective dynamics in network models, however, has proved challenging (Ahmadian 2021). Resolving this challenge will be crucial…

- The lines following our introduction of the classic balanced state model now describe the relationship between E-I balance and asynchronous irregular activity, while also noting the Reviewer's comment that E-I balance and asynchronous irregular activity do not always coincide (LINES 21-27):

  "In this [classic balanced state] model excitatory-inhibitory balance is central to irregular activity as excess inhibition or excitation lead to quiescence or regular (non-Poissonian) spiking, respectively. (Note that excitatory-inhibitory balance can also play a key role in networks that do not produce irregular spikes (Ingrosso 2019; Kadmon 2020; Engelken 2022), and irregular spiking can emerge without balance, e.g. when driving neurons with external input noise; however, most spiking models of cortex assume that balance and irregular activity are tightly linked (Ahmadian 2021).)"

- In the final paragraph of the introduction, we have added an explicit description of what we mean by "balance" in the network of WTA units proposed in this study (LINES 61-72):

  We refer to this dynamical regime as the balanced state of the network [of WTA units], in analogy with the classic excitatory-inhibitory balanced state (van Vreeswijk 1996; van Vreeswijk 1998; Brunel 2000; Ahmadian 2021). In the network of WTA units, however, balance exists not between excitatory and inhibitory populations but among multiple populations of neurons with different tunings---hence the balance is ``multi-dimensional.'' Individual WTA units receive effectively random, yet statistically balanced inputs from all active populations; but within a unit only one neuron can spike at each time step, resulting in Poisson-like activation patterns. Crucially, the multi-dimensional nature of this balance allows flexible, nonlinear collective dynamics to unfold simultaneous with and sustained by consistent Poisson-like spiking activity. Thus, networks of WTA units can support fluctuation-driven irregular activity capable of powering flexible nonlinear collective dynamics.

We have also removed the phrase "generalization of the classic balanced state", as we agree with the Reviewer that this may be confusing.

We hope that the above changes will clarify the relationship between asynchronous irregular activity and E-I balance, as well as what we mean by balance in our network model.

2) Line 100: It might be more accurate to change the two appearances of the work "any" to "all"?

We have changed "all" to "any" in this line. (We assume the Reviewer was suggesting "all" → "any", since the original line used the word "all").

3) Lines 127-133: It might be worth mentioning or at least citing the work of Monteforte and Wolf here:

Monteforte, Michael, and Fred Wolf. "Dynamic flux tubes form reservoirs of stability in neuronal circuits." Physical Review X 2.4 (2012): 041007.

Monteforte, Michael, and Fred Wolf. "Dynamical entropy production in spiking neuron networks in the balanced state." Physical review letters 105.26 (2010): 268104.

Engelken, Rainer, Michael Monteforte, and Fred Wolf. "Sparse chaos in cortical circuits." arXiv preprint arXiv:2412.21188 (2024).

They studied similar notions of "effective" chaos. The idea, I think, is that the network is not strictly speaking chaotic for fixed N, but becomes chaotic as N goes to infinity (though it's effectively chaotic in practice for relatively small N). I imagine something similar is happening in these models where a finite state space prevents true chaos, but as the state space grows, the dynamics approximate chaos.

Thank you for the references. These studies indeed show that irregular spiking does not strictly imply chaos (as defined via Lyapunov exponents). For instance, Monteforte & Wolf 2012 show how one can find small perturbations that do not lead to exponentially diverging state trajectories (corresponding to a negative maximum Lyapunov exponent), even though single-spike perturbations diverge, hence the networks are effectively chaotic.

We have now added the suggested citations to the line in question, as well as Jahnke, Memmesheimer, Timme, PRL 2008, ("Stable Irregular Dynamics in Complex Neural Networks"), who show that highly irregular dynamics can coexist with stable periodic orbits, which is closer to the situation in the finite-size network of WTA units. The line in question now reads (LINES 153-158):

> Note that strictly speaking for finite N the $g \to \infty$ limit is not chaotic, since the dynamics unfold deterministically on a discrete space so must eventually fall into a periodic orbit; however, they are effectively chaotic, as measured by sensitivity to small perturbations (Fig S1), and we expect them to approximate true chaos more closely as $N \to \infty$ (Jahnke 2008; Monteforte 2010; Monteforte 2012; Engelken 2024).

Note that we have also added a supplemental figure numerically demonstrating the sensitivity of microscopic (spiking) trajectories following a single spike-swap perturbation, for the unstructured/uniform network and similarly for the multistable and sequence-generating network (S1,S4,S7 Fig).

A further interesting note about the WTA network proposed in this study is that if one considers the inputs as dynamical variables, then we in fact expect the network to exhibit similar "flux tubes" as those described in

Monteforte & Wolf 2012. This is because many microscopic perturbations to these inputs won't change which neuron in each unit spikes (hence the dynamics are stable with respect to such perturbations), even though high sensitivity still exists to spike perturbations. However, we would like to reserve a more thorough analysis of this idea for future work.

4) Lines 227-229: When discussing the linearity/nonlinearity of rates in the balanced state, it might be worth citing Zhu, Baker, and Rosenbaum:
Baker, Cody, Vicky Zhu, and Robert Rosenbaum. "Nonlinear stimulus representations in neural circuits with approximate excitatory-inhibitory balance." PLoS computational biology 16.9 (2020): e1008192.

Thank you for the suggestion. We have added the following clause to the line in question (LINES 260-261):

> "although nonlinear responses can emerge if populations are allowed to be silenced via excess inhibition (``semi-balance'') (Baker 2020)."

5) Lines 271-275: When discussing multistability in balanced networks, it could be worth citing the same paper by Zhu et al, in which multiple stable fixed points can arise in a generalization of balanced networks.

Thank you for the suggestion. We have added the citation of Baker, Zhu, Rosenbaum 2020 to this line (LINES 309-310):

> Multistability can also emerge in semi-balanced networks, in which certain populations are allowed to be silent (Baker 2020).

6) For many equations in the manuscript (e.g., Eq 2), the text following the equation is indented even though there is no paragraph break. The authors can avoid this by not skipping a line in their TeX code after the equation.

Thank you for noting this. We have fixed all of the indentations.

General discussion/comments that the authors are free to ignore:

1) General comment (pertaining in part to Lines 577-592): The authors discuss some predictions and interpretations of their results for neural circuits. It seems their model also predicts the presence of co-tuned populations of neurons. Of course, co-tuned neurons exist in neural circuits. But do these models make any additional predictions about the shared properties or dynamics of co-tuned populations beyond just similar preferred stimuli?

Thank you for the comment. One of the main predictions of the model, beyond the existence of co-tuned neurons, is that the functional couplings ($J_{ij}^{dd'}$s in the model) between co-tuned neurons will have higher *variance* than between non-co-tuned neurons, i.e. $\mathrm{Var}(J_{ij}^{dd}) > \mathrm{Var}(J_{ij}^{dd'})$ for $d \neq d'$. This stands in contrast to the common idea that co-tuned neurons should have increased *mean* couplings (although we do not rule this out). The idea underpinning the latter is that higher mean connectivity will lead these neurons to reinforce one another, changing tuning properties and potentially leading to persistent activation (even absent a stimulus) if the connectivity is strong enough. In the network model we have proposed, however, reinforcement between co-tuned neurons emerges through higher *variance* functional couplings. The clearest demonstration is in the multistable network, where all mean weights are 0 and the increased weight variances within co-tuned populations leads to the within-tuning feedback (while the WTA induces competition among tunings), which stabilizes the macroscopic network state. Thus, the main prediction of the model is that co-tuned neurons

should have higher variance functional couplings. This is clarified in our Discussion section on Predictions (LINES 675-680):

> "...we predict that neurons with correlated slow changes in firing rates (i.e. with similar ``tuning") should have higher *variance* functional couplings at millisecond timescales, but not necessarily higher mean couplings, as in our model the former are the source of slow timescales. This could be tested by fitting a network-generalized-linear-model (Pillow 2008) to population spike trains and comparing the inferred coupling filters between neuron pairs with the strength of correlation of their firing rates."

We have also added a further prediction of the model in this paragraph, which is essentially that the firing rate dynamics described by the mean-field equations for $\mathbf{r}^t$ in our study will provide a better description of firing rate dynamics than traditional models, e.g. of the form $\tau d\mathbf{h}/dt = \mathbf{-h} + W\Phi(\mathbf{h}) + \mathbf{u}$, where Φ is a nonlinearity (LINES 688-694):

> Our final core prediction is that empirical neural dynamics will be better described by a class of rate equations closer to our mean-field equations (Eq 5-10) than the more common model class of the form $\tau d\mathbf{h}/dt = \mathbf{-h} + W\Diamond(\mathbf{h}) + \mathbf{u}$, where $\mathbf{r} = \phi(\mathbf{h})$ are the firing rates and ϕ is an element-wise monotonic nonlinearity (Sompolinsky 1988; Sussillo 2009; Mastrogiuseppe 2018; Engelken 2022; Driscoll 2024; Schuessler 2024). This could be tested by fitting both models to data, treating the firing rates as latent variables that produce observed spikes through a Poisson process, and comparing the fits.

As a related but separate question, how might this model be consistent with the presence of complex cells whose response properties are not described by a single preferred stimulus? These are open-ended questions/suggestions that the authors are free to ignore and save for future work.

Thank you for the question. Extending the model to incorporate overlapping tunings or multiple preferred stimuli is one of the key next steps we plan to undertake, especially since this will likely make the model more amenable to fitting to empirical data where neurons have overlapping stimulus preferences.

For the time being, a brief note is that in the present model the stimulus tunings per se do not matter that much in terms of producing nonlinear behavior. For instance, while stimuli can be used to set the initial state of the multistable or sequence generating network, the sustained network dynamics (staying in the initial state or evolving sequentially) unfold with no stimulus given. Instead, the central component underlying the nonlinear behavior of the network is that neurons within each WTA unit have different "types" (1, …, D), where *d* means the same thing in each unit. Associating these types with stimulus preference or tuning was a useful abstraction, but it is not necessarily the only one. For instance, the "types" within each unit could also be defined first and foremost by their functional connectivity or co-activation patterns, without necessarily referencing any stimulus in the first place (e.g. in deeper brain areas). Similarly, multiple "types" of neurons could have the same stimulus tuning—this would change the input response of the network but not the autonomous nonlinear behavior.

In any case, this is certainly an interesting question that we would like to explore in more detail in future work. For the present we have noted this as a future direction in our Discussion section (LINES 660-664):

> It will be particularly useful to examine robustness against perturbations of the competitive nonlinearity, e.g. if competition is not symmetric among neurons in a unit but spreads across units, possibly asymmetrically; if not every unit contains every tuning; or if neurons have continuous and/or overlapping tunings rather than discrete tunings.

2) General comment (pertaining to Lines 497-518): The authors discuss some interpretations of their model in terms of neural circuits (e.g., lateral inhibition). An additional interpretation is that each "neuron" in their models might represent something more abstract in a real neural circuits, e.g., a linear projection of neural activity in a population in some other direction (as in a distributed code under WTA dynamics). If the authors want, they could discuss this possibility, but this is a completely optional suggestion.

Thank you for the interesting suggestion. We have added this point to the Biological Basis section of the Discussion (LINES 586-589):

> In principle, competition within a unit could also occur between different activity patterns rather than different neurons, with these patterns competing in a WTA-like fashion; or similarly between attractor-like states in small cortical patches (Ryom 2023).

Reviewer #2:
This is a very interesting paper.

The author proposes a model network that reconciles temporal irregular activity at the microscopic level with non-linear dynamics at the macroscopic level. Non-linear dynamics is clearly a sine qua non for computation. On the other hand, temporal irregularity is an experimental constraint that any suitable model/theory of cortical function must reproduce. The standard account of temporal irregularity is the theory of balanced networks that in its common implementation, however, does not produce non-linear dynamics at the macroscopic level (e.g., multi-stability). Essentially, this is because the synaptic input driving the activity of a neuron is a linear function of the pre-synaptic 'firing rates'. In the present contribution, this linearity is 'destroyed' by the winner-take-all (WTA) mechanism within a 'unit'; de facto, the activity of a given neuron depends not only on its own synaptic input but on the synaptic inputs to all the neurons in the same unit.

In the manuscript, the author illustrates the different dynamical regimes that such networks can exhibit and develops an accompanying mean-field theory that is shown to well capture some quantitative aspects of the relevant dynamics. The paper is well written. The results are novel to the best of my knowledge and, as I have already said, interesting. I have a few minor comments that mostly amount to the clarification of a few points.

Thank you very much for the comments and accurate summary of the study.

I was confused by the section "Heterogeneous fluctuation-driven firing rate dynamics". Basically, I didn't understand what the author is trying to achieve.

Thank you for the comment. We apologize for the confusion and have now rewritten this section to more clearly describe our motivation and results (copied in magenta below).

Briefly, the goal of this section is to show that the firing rate dynamics, i.e. the dynamics of the D-dimensional firing rate vector $\mathbf{r}^t$, can exhibit more complex temporal patterns than multistability and sequences alone. The mean-field description of these dynamics, derived as the $N \rightarrow \infty$ limit of the full spiking dynamics, yields the time-evolution equations:

$$r_d^t \approx \int_x \mathcal{N}\left(x; \mu_d, \sigma_d^2\right) \prod_{d' \neq d} \Phi\left(x; \mu_{d'}, \sigma_{d'}^2\right) dx \quad (5)$$

$$\mu_d \approx F_\mu\left[\mathbf{r}^{t-1}\right] \equiv D\sum_{d'}\mu_J(d,d')r_{d'}^{t-1} + u_d^t \quad (9)$$

$$\sigma_d^2 \approx F_\sigma\left[\mathbf{r}^{t-1}\right] \equiv D\sum_{d'}\sigma_J^2(d,d')r_{d'}^{t-1} + v_d^t \quad (10)$$

Specifically, these equations give $\mathbf{r}^t$ in terms of $\mathbf{r}^{t-1}$ and the network parameters D, μ_J, and σ_J^2 (μ_J and σ_J^2 are both DxD matrices), as well as the input means and variances u_d and v_d (both zero in this part of the study). We emphasize that these dynamics, which are for the D-dimensional vector $\mathbf{r}^t$, are distinct from the ND-dimensional spiking dynamics $\{\mathbf{y}_i^t\}$, with the former constituting the mean-field description of the latter.

In the previous section we showed how to program Eq 5, 9, 10 (i.e. by choosing an appropriate matrix σ_J^2) to produce multistability and sequences in the mean-field dynamics for $\mathbf{r}^t$. In this section, we show how to choose σ_J^2 to produce much more complex, heterogeneous, time-varying firing rate dynamics, e.g. (from Fig 8b):

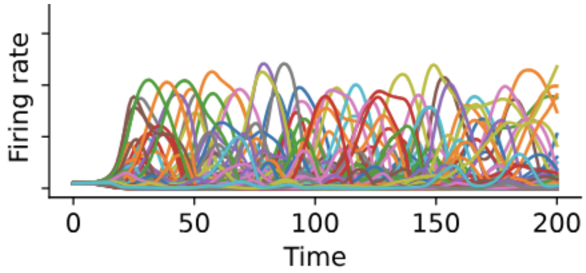

Further, up until this simulation, it was not clear whether one could achieve both irregular “microscopic” spiking simultaneous with heterogeneous, time-varying “macroscopic” firing rates. For instance, the simulations of the unstructured network in Fig 1-2 produce irregular spikes, but the firing rates $\mathbf{r}^t$ are constant.

In the figure above, however, we show that these two phenomena can co-exist—complex, time-varying macroscopic firing rates can coexist with Poisson-like irregular spiking in the model. Intuitively, this is because (1) the complex firing rate dynamics are a consequence of the dynamical system (specified by Eq. 5, 9, 10 above) parameterized by a random, sparse, (and strictly positive) matrix σ_J^2; and (2) the irregular spiking in the full/microscopic spiking model emerges because we have implemented this simulation by exclusively adjusting the matrix σ_J^2, which corresponds to the network operating in a purely fluctuation-driven regime.

Clearly it is not some chaotic dynamics a la Sompolinsky-Crisanti-Sommers (though this is one of the references cited), because this has already been demonstrated in Fig. 1.

The comparison to Sompolinsky et al 1988 and other rate networks refers to the fact that typically complex firing rate dynamics are studied as evolving according to a different set of equations—usually of the form $\tau d\mathbf{h}/dt = -\mathbf{h} + W\mathbf{r} + \mathbf{u}$ with $\mathbf{r} = \phi(\mathbf{h})$, where ϕ is a nonlinearity. For instance, Sompolinsky et al 1988 showed how such a network could produce rate chaos, corresponding to strong temporal variability in $\mathbf{r}$, with subsequent studies (e.g. Sussillo & Abbott 2009) showing how such dynamics could advantage learning.

In our study we show that our equations for $\mathbf{r}^t$ (Eq. 5, 9, 10) can also produce highly temporally variable dynamics, even though they have a very different form from the classic SCS network. As mentioned above, since these dynamics correspond to a fluctuation-driven regime of the full spiking network, they also co-exist with irregular spiking. A natural extension will be to ask whether our equations for $\mathbf{r}^t$ can also produce chaos (or if the dynamics are just long transients), but we would like to reserve a rigorous investigation of this for future work.

We now clarify the motivation and results of this section via several updates to the manuscript text:

- First, to prevent any confusion between the microscopic spiking dynamics and the macroscopic firing rate dynamics (derived as the mean-field description of the former), we have added the following paragraph to our "Terminology" section of our model description (LINES 94-101):

  Our study also involves two classes of dynamical systems, which we will frequently compare. The first class (Eq 1-4 below) describes the ``microscopic'' activity dynamics of each neuron in each WTA unit, which we will often refer to as the *full* or *full spiking* (in the high-gain limit) dynamics. The second class (Eq 5-10 below) describes the dynamics of the ``macroscopic'' firing rates $\mathbf{r}^t \in R^D$ of the D populations, where the firing rate of a population is the average spiking activity of the N neurons in that population at time t---these dynamics are derived as a mean-field description of the full spiking dynamics, and which we will refer to as the *mean-field* dynamics for $\mathbf{r}^t$.

- Second, for further clarification, in introducing the simulations of structured networks we have added the lines (LINES 297-299):

  In each setting, we first study the mean-field dynamics (Eq 5-10) of the D-dimensional vector $\mathbf{r}^t$ of firing rates of the D populations, then corroborate our results in the corresponding full spiking network (Eq 1-4) governing the dynamics of the single-neuron activities $\{\mathbf{y}_i^t\}$.

- Third, addressing the Reviewer's point most directly, we have changed the start of the final section, "Heterogeneous fluctuation-driven firing rate dynamics," to clarify our motivation and approach, which now reads (LINES 443-477):

  Finally, we asked whether the network could produce more complex firing rate dynamics $\mathbf{r}^t$ than multistability and sequences alone, yet still simultaneous with irregular spiking. Complex, time-varying firing rates are thought to provide a reservoir of temporal patterns that can be composed into flexible computations (Jaeger 2001; Sussillo 2009; Abbott 2016; Huang 2017; Driscoll 2024). Typically, however, such dynamics are studied in ``rate network'' models of neural populations that do not explicitly account for spikes; most commonly, a D-dimensional vector of population firing rates, $\mathbf{r} = \phi(\mathbf{h})$, evolves according to $\tau d\mathbf{h}/dt = -\mathbf{h} + W\mathbf{r} + \mathbf{u}$, where $\mathbf{h}$ are private variables, $\mathbf{u}$ are inputs, τ is a time constant, W is a D x D matrix of interaction weights between populations, and ϕ is an element-wise nonlinearity such as a sigmoid (Sompolinsky 1988; Sussillo 2009; Mastrogiuseppe 2018; Engelken 2022; Driscoll 2024; Schuessler 2024). In contrast, our equations for $\mathbf{r}^t$ (Eq 5-10), which also describe the time-varying firing rates of D populations, operate fundamentally differently—besides being in discrete time, Eq 5-10 are parameterized by two DxD matrices, μ_J and σ_J^2, and are mediated by a *non*-element-wise nonlinearity (Eq 5). Thus, it is unclear how similarly they behave to the more common rate network equations. Note also that while the equations for $\mathbf{r}^t$ are derived (as the mean-field description of the full network dynamics) under the assumption of irregular spiking in $\{\mathbf{y}_i^t\}$, irregular single-neuron activity does not imply irregular or complex dynamics in $\mathbf{r}^t$; for instance, in the unstructured network the spikes are irregular but the population firing rates are constant (Fig 2-3). Here, we investigate whether irregular activity in $\{\mathbf{y}_i^t\}$ can co-exist with complex, time-varying dynamics of the population firing rates $\mathbf{r}^t$.

  To address this problem, we simulated the mean-field equations for $\mathbf{r}^t$ (Eq. 5, 9, 10; $N\to\infty$ limit), fixing $\mu_J=0$ and only allowing ourselves to vary the matrix parameter σ_J^2. This procedure keeps the network in the fluctuation-driven regime (since all mean inputs are 0; Eq. 6, 9), which is in turn expected to

produce irregular spiking in the corresponding full simulations. A challenge of this approach, however, is that whereas the matrix W in classic rate networks can usually contain both positive and negative values (Sompolinsky 1988; Sussillo 2009; Mastrogiuseppe 2018; Driscoll 2024; Schuessler 2024), all elements of σ_J^2 are non-negative by definition. (Note that in our full model, the weights $J_{ij}^{dd'}$ between individual neurons can be both positive and negative [Eq 4]---the non-negative matrix σ_J^2 specifies the variances of $J_{ij}^{dd'}$, in both the mean-field equations for $\mathbf{r}^t$ and the full model, and is the only parameter we vary in the present simulations of $\mathbf{r}^t$.) Given this constraint, it is not obvious that Eq 5-10 could produce activity with the same level of complexity as can arise in classic rate networks. We hypothesized, however, that if σ_J^2 were a sparse random matrix, the normalization of the network activity through Eq. 5 would play a similar role as the inhibitory feedback in classic rate networks…

We hope that this rewriting clarifies the aim and approach of this section.

Is it some meta-stability as in refs 23 and 24? If so, this should be clearly stated, possibly citing the relevant references. Also, if so, it seems that the proposed solution does not solve the basic problem of refs 23-24; that is, metastability is essentially a finite-size effect (here, controlled by the parameter K_\sigma).

Thank you for pointing out this connection. Producing metastability in the network was not our goal per se (see above); however, as suggested by the Reviewer the network dynamics do have a connection to previous studies of metastability (e.g. Litwin-Kumar & Doiron 2012 and Mazzucato et al 2015), in which the slow timescales come from within-cluster positive feedback and the transitions from fluctuations due to finite-size effects.

In our network, in certain instantiations of the rate/mean-field equations (e.g. below; Fig 8b) one can clearly see that certain populations (tunings) are active for many timesteps (recall that the single neuron/WTA unit update equations from which the MFT is derived have a timestep of 1):

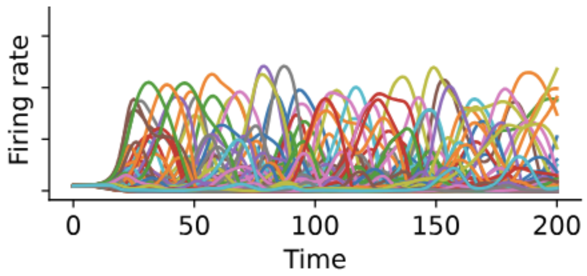

As these simulations correspond to the N→∞ mean-field approximation of the spiking dynamics, these “slow” timescales presumably do not come from finite-size effects in N. However, in the full spiking network simulations, finite-size effects may play a role in changing the collective network state. We discuss this in our revised manuscript near the end of the section in question (LINES 515-522)

> Note that in the full spiking simulations some of the dynamics may arise from finite-size effects, similar to how finite numbers of neurons in previously studied balanced clustered networks allow spontaneous fluctuations to drive transitions between metastable states (Litwin-Kumar 2012; Mazzucato 2015). However, because slow timescales persist in our mean-field simulations (computed in the N→∞ limit), the slowness and complexity of the firing rate dynamics are likely not exclusively a finite-size effect in N. Intuitively, the slowness is inherited from the multistable network, with the additional couplings between the mean-field populations via the nonzero entries of σ_J^2 destabilizing the multistability.

In the same section, lines 418-426. This is confusing. In the network, synaptic interactions (i.e., the J's of Eq. (4)) are both negative and positive, though their variances are obviously positive. Clearly, you must be talking

about the "interactions" among the mean-field populations (i.e., Eqs. (5), (9) and (10)). See also my previous comment.

We apologize for the confusion. The Reviewer is correct that this statement refers to interactions of the mean-field populations. We have clarified this with the following parenthetical (LINES 469-473):

> (Note that in our full model, the weights $J_{ij}^{dd'}$ between individual neurons can be both positive and negative [Eq 4]---the non-negative matrix σ_J^2 specifies the variances of $J_{ij}^{dd'}$, in both the mean-field equations for $\mathbf{r}^t$ and the full model, and is the only parameter we vary in the present simulations of $\mathbf{r}^t$.)

The author carefully discusses the possible biological mechanisms underlying the WTA mechanism. It is not clear, however, that any of these mechanisms (or even a combination of them) would lead to a "perfect" WTA as the one used in the theory and simulations. As it is implemented now the mechanism is 'fiercely' non-local (despite the author calling it 'local' several times); the activity of a neuron depend on the same-time inputs to other neurons, i.e., it is non-local in time. So, it seems to me, there is also an issue of time scales that would require a short discussion; the time scale over which the WTA is achieved vs. the time scale of the synaptic interaction between the different units.

Thank you for the comment. We apologize for our imprecise usage of the term “local”. The Reviewer is correct that the WTA interaction as implemented is non-local in time since each neuron has access to the other neurons’ presynaptic inputs at the same timestep. We have now replaced “local” with other terms, e.g. “within-unit” in several places in the text to avoid confusion. To address the issue of timescales explicitly we have also added the following lines to the Discussion on biological plausibility (LINES 588-597):

> An important consideration for more detailed network models is the timescales over which the key interactions unfold. In particular, we have modeled the integration of presynaptic inputs and the WTA operation as occurring within one timestep, roughly corresponding to one membrane time constant (10-20 ms [Kandel 2000]). Indeed, neurons integrate their presynaptic inputs over this timescale (Kandel 2000), and it has been shown that WTA-like operations can occur at or faster than this timescale in small networks of integrate-and-fire model neurons (Jin 2002), suggesting the fast timescales in our model are consistent with biology. Formally extending our network to operate with continuous-time integrate-and-fire neurons that compete within small groups while also receiving fluctuating inputs will be important future work.

As an historical aside, the same mechanism was considered by Bienestock ('A model of cortex', 1995) with motivations partially overlapping with the ones behind the present work. In particular, he introduces a dynamics similar to the one studied here and faces more or less the same problems in attempting a 'biological justification'.

Thank you for the interesting reference. We have now added the following line/citation when introducing the WTA component of our network model (LINE 125):

> Constant WTA-mediated activity, as a functional consequence of inhibitory feedback, has also been proposed in a ``synfire'' model of cortical dynamics (Bienenstock 1995).

Understanding how WTA-like functionality could emerge from more biologically realistic synaptic interactions will indeed be an important avenue for future work.

Finally, perhaps I'm too old and my eyesight is not as good as it used to be, but figures are really small. A simple solution would be to reduce the number of sub-figures within a 'figure'. Obviously, the author is free to ignore this suggestion.

We apologize for the small text and difficulty in reading the figures. We have now increased the font sizes for many of the figure labels, as well as split the three bulkiest figures (multistability, sequences, heterogeneous firing rate dynamics) into two figures each, with the first showing examples and the second showing additional statistics/analyses.

Reviewer #3:

In this paper, Pang analyzes a network of spiking neurons incorporating a winner-take-all mechanism. By studying the network dynamics in the mean-field limit, the work demonstrates that such networks can exhibit a fluctuation-driven regime supporting flexible, nonlinear firing rate dynamics. These findings are further substantiated through simulations of the microscopic model. The observed behavior is attributed to the modulation of synaptic weight variance between distinct neuronal blocks.

The study is well-written, the mathematical derivations are robust, and the biological plausibility of the mechanism is appropriately discussed in the manuscript.

While alternative mechanisms capable of generating nonlinear rate dynamics exist and the model's units are not biologically realistic - as acknowledged by the author - I believe this work remains valuable and relevant to the research community.

Thank you for the comment and for the accurate summary of the study. Please note that in response to R2's suggestions, we have also added further details about possible biological implementations of the WTA units, in particular addressing the timescales over which the synaptic and WTA interactions would occur in a more realistic network composed of leaky integrate-and-fire neurons.

My main concern lies with the manuscript's mention of "computational" properties. While I agree that nonlinear dynamics, multistability, and sequential activation are fundamental building blocks of computational capabilities, no explicit computations are presented in this manuscript. Although it is reasonable to expect that the network could perform tasks similar to other rate models, this is not explicitly shown here. As such, I suggest framing the references to computational properties in a more conditional manner, for example, by emphasizing the potential for such applications rather than asserting them outright, particularly in the abstract (second-to-last paragraph) and the author summary (last two paragraphs).

Thank you for the suggestion. The Reviewer is correct that the simulations in the paper primarily demonstrate dynamical behavior and not computations per se. For clarity we have now updated the final lines of the abstract to instead use the phrase "well posed for flexible computation":

> Thus, networks of WTA units support a chaotic fluctuation-driven regime characterized by irregular spiking that can power complex nonlinear collective dynamics. This represents a new model of brain activity capable of simultaneously reproducing realistic spike trains and diverse nonlinear firing rate patterns well posed for flexible computation, and which can be trained or fit to data.

Similarly, we have updated the second half of the author summary to:

Here we present a network model, based on winner-take-all units akin to small groups of neurons, in which irregular spiking and flexible nonlinear dynamics go hand-in-hand and can be related via a concise mathematical theory. The result is a new model of brain activity capable of simultaneously capturing realistic spiking and firing rate dynamics, which is well posed for flexible computation and can be trained or fit to data.

We also note that investigating more interesting computations by the proposed network (e.g. Hopfield-like pattern completion) is one of the primary future directions for this work. In particular, the (mean-field) dynamics for the firing rate vector $\mathbf{r}^t$ have a substantially different form from traditional RNN equations, and we are excited to investigate whether they may exhibit any specific advantages as a substrate for computation through dynamics (e.g. since the neurons tend to stay away from saturation due to the WTA interaction).

Other suggestions:

- An alternative mechanism for the generation of nonlinear dynamics in balanced networks has been proposed by Sanzeni, Histed, and Brunel (2020) in Response nonlinearities in networks of spiking neurons (PLoS Computational Biology). They show that while a linear transfer function is obtained in the strong coupling limit, nonlinearities emerge at response-onset and saturation as the coupling strength decreases. The author might consider discussing why a winner-take-all mechanism is necessary instead of this approach (maybe related to the finite-size theories mentioned in line 27?)

Thank you for the interesting reference. We now discuss the relationship between our model and finite-size theories, including both “loose balance” and the Sanzeni, Histed, Brunel 2020 study, in our updated Discussion (LINES 552-561):

> Our mean-field equations for the firing rates $\mathbf{r}^t$ describe deterministic, nonlinear dynamics even in the $N \to \infty$ limit (Eq. 5, 9-10). Thus, the network's nonlinear behavior holds when neurons receive an infinite number of synaptic inputs. This contrasts with finite-size theories, e.g. the theory of ``loose balance'', which reproduces empirical nonlinear phenomena as a consequence of neurons receiving finite synaptic inputs (Ahmadian 2013; Rubin 2015; Ahmadian 2021), yet which converges to the linear behavior of the classic balanced state when synaptic inputs are sufficiently numerous. A related model (Sanzeni 2020) showed how single-neuron response-onset and saturation nonlinearities could partially propagate to the network level if recurrent interactions were weak, but this network also relied on finite synaptic inputs or weak couplings.

- line 488-489: The author might consider comparing the proposed approach to the work of Recanatesi et al. (2022) in Metastable attractors explain the variable timing of stable behavioral action sequences (Neuron). In this study, transitions between attractors are driven by low-dimensional correlated variability in the input, which could provide a useful perspective for generating more complex temporal dynamics in balanced networks.

Thank you for the suggestion. Exploring what factors might govern the temporal variability of the sequences produced by our proposed network, in particular to match that observed in behavioral data, is a very interesting idea, and the role of correlated variability proposed in Recanatesi et al 2022 will be an excellent starting point. We now discuss this in the second paragraph of our revised Discussion (LINES 572-578):

> An alternative model showed how correlated noise could drive sequences of state transitions matching empirical temporal variability (Recanatesi 2022), but this network did not include spikes---it will be interesting to explore whether correlated noise can facilitate dynamics in our network as well. In general, it will be interesting to compare these and other balanced network models (Vogels 2009; Vogels 2011;

Mongillo 2012; Boerlin 2013; Kraynyukova 2018; Baker 2020; Sanzeni 2020; Ahmadian 2021; Khajeh 2022; Podlaski 2024) with our network in terms of the collective dynamics they can produce and their relationship to empirical neural dynamics.

- Another important point concerns the figure captions, which are somewhat difficult to read due to their small size. This may be related to the images not spanning the full page width. Enhancing the caption size and ensuring figures are appropriately scaled would improve the manuscript's clarity.

We apologize for the difficult-to-read text in the figures. We have now increased the font sizes for many of the figure panels, as well as split the three largest figures (previously Fig 4: multistability, Fig 5: sequences, and Fig 6: heterogeneous firing rate dynamics) into two figures each, with the first figure showing examples and the second statistics/further theoretical analyses. We expect that they should be substantially easier to read now.
